# Prevention by the CXCR2 antagonist SCH527123 of the calcification of porcine heart valve cusps implanted subcutaneously in rats

**DOI:** 10.3389/fcvm.2023.1227589

**Published:** 2023-09-15

**Authors:** Yuthiline Chabry, Kawthar Dhayni, Saïd Kamel, Thierry Caus, Youssef Bennis

**Affiliations:** ^1^ MP3CV Laboratory, UR UPJV 7517, Amiens, France; ^2^ Department of Cardiac Surgery, Bichat Hospital, Paris, France; ^3^ LVTS unit, INSERM, Paris, France; ^4^ Department of Clinical Biochemistry, CHU Amiens-Picardie, Amiens, France; ^5^ Department of Cardiac Surgery, CHU Amiens-Picardie, Amiens, France; ^6^ Department of Clinical Pharmacology, CHU Amiens-Picardie, Amiens, France

**Keywords:** aortic valve replacement, bioprosthesis, structural valve deterioration, calcification, inflammation, chemokines

## Abstract

**Introduction:**

Calcification is a main cause of bioprosthetic heart valves failure. It may be promoted by the inflammation developed in the glutaraldehyde (GA)-fixed cusps of the bioprosthesis. We tested the hypothesis that antagonizing the C-X-C chemokines receptor 2 (CXCR2) may prevent the calcification of GA-fixed porcine aortic valves.

**Materiel and methods:**

Four-week-old Sprague Dawley males were transplanted with 2 aortic valve cusps isolated from independent pigs and implanted into the dorsal wall. Four groups of 6 rats were compared: rats transplanted with GA-free or GA-fixed cusps and rats transplanted with GA-fixed cusps and treated with 1 mg/kg/day SCH5217123 (a CXCR2 antagonist) intraperitoneally (IP) or subcutaneously (SC) around the xenograft, for 14 days. Then, rats underwent blood count before xenografts have been explanted for histology and biochemistry analyses.

**Results:**

A strong calcification of the xenografts was induced by GA pre-incubation. However, we observed a significant decrease in this effect in rats treated with SCH527123 IP or SC. Implantation of GA-fixed cusps was associated with a significant increase in the white blood cell count, an effect that was significantly prevented by SCH527123. In addition, the expression of the CD3, CD68 and CXCR2 markers was reduced in the GA-fixed cusps explanted from rats treated with SCH527123 as compared to those explanted from non-treated rats.

**Conclusion:**

The calcification of GA-fixed porcine aortic valve cusps implanted subcutaneously in rats was significantly prevented by antagonizing CXCR2 with SCH527123. This effect may partly result from an inhibition of the GA-induced infiltration of T-cells and macrophages into the xenograft.

## 1. Introduction

Surgical or transcatheter aortic valve replacement (SAVR or TAVR) is currently the only effective curative treatment of aortic valve stenosis. The biological valves made from porcine or bovine tissue are the most commonly used valve substitutes today (1). Indeed, compared to mechanical valves, bioprostheses have a low risk of thrombosis and do not require lifelong anticoagulant treatment (2, 3). However, they are prone to failure. According to the criteria of the VARC-3 consortium (4), it is now possible to differentiate the etiologies of biological prosthesis failure and to diagnose an early stage of structural valve deterioration (SVD), before the ultimate stage of reoperation. SVD corresponds to a permanent intrinsic alteration of the valve leaflets responsible for progressive stenosis and/or hemodynamic leakage of the bioprosthesis. In the recent observational SAPIEN 3 registry, the 5-year cumulative rate of SVD-related hemodynamic valve deterioration or valve failure was 3.5% by SAVR (among 664 patients) and 3.9% by TAVR (among 891 patients) over the same time frame (5). Calcification of the bioprosthetic leaflets is a main feature of SVD and has been strongly associated with hemodynamic and clinical outcomes, including the need for reoperation and death (6). Although the mechanisms are not fully understood, the calcification can be related to the fixation process used to manufacture and store bioprostheses. Indeed, porcine valve cusps or bovine pericardium are fixed with glutaraldehyde (GA) that induces cross-linking of collagen fibers and devitalizes the tissues, making them rot-proof during their conservation, and tanning them, which reinforces their resistance to mechanical stresses (7). However, GA promotes calcification by disrupting cell membranes, which increases the influx of calcium and decreases its efflux due to a loss of activity of the membrane pumps (8). In addition, it has been reported that the GA cross-linking does not completely eliminate the antigenicity of xenogeneic tissues; hence an immune response can take place on the bioprosthesis, which leads to the infiltration of macrophages, monocytes and T cells, and the development of local inflammation (9, 10). The breakdown products of the inflammatory response lead to apoptotic bodies, extracellular vesicles, and other debris, which are promoters of dystrophic calcification (11). Chemokines and chemokine receptors may play a critical role in the recruitment of inflammatory cells into the bioprosthesic valve and thus in their calcification (12). Therefore, using a model of subcutaneous porcine aortic valve implantation in rats, we tested the hypothesis that antagonizing the C-X-C chemokine receptor 2 reduces *in vivo* the calcification of GA-fixed porcine aortic valves.

## 2. Materials and methods

### 2.1. Collection of porcine aortic valves and GA pre-incubation

Hearts were isolated from pigs in a slaughter house (Bigard, Saint-Paul-sur-Ternoise, France) and dissected within up to 4 h to harvest aortic valvular cusps. Then, aortic cusp pieces of 25 mm^2^ were incubated for 12 h at 4°C in a 30 mM HEPES solution [acid 4-(2-hydroxyethyl)-1piperazine ethane sulfonic, ref. c-40020, PromoCell], with or without 0.6% GA (ref. G5882, Sigma Aldrich).

### 2.2. Animals and subcutaneous implantation of the valvular cusps

Twenty four 4-week Sprague-Dawley male rats were used as hosts, after 1 week acclimation in the animal platform PLATANN of the University of Picardie Jules Verne, Amiens, France (agreement B80021009). Surgery was performed under general anesthetic (isoflurane gas). Rats were in prone position. Two incisions were made on the back of the host, then soft dissection with scissors permitted to obtain pouch for each incision. Aortic valvular cusps were rinsed in sterile 0.9% sodium chloride solution and then put inside of the pouch. The origin of the 2 pieces of valve cusps implanted in the same rats came from different pigs. The incision was then closed by separate points of prolen 5.0 and a local analgesic was injected all around the incision. This experimental project was authorized under the reference APAFIS#1945-2019022212357854 v4, in accordance with the European Directive 2010/63/EU.

### 2.3. Rat treatments

**Figure 1:**
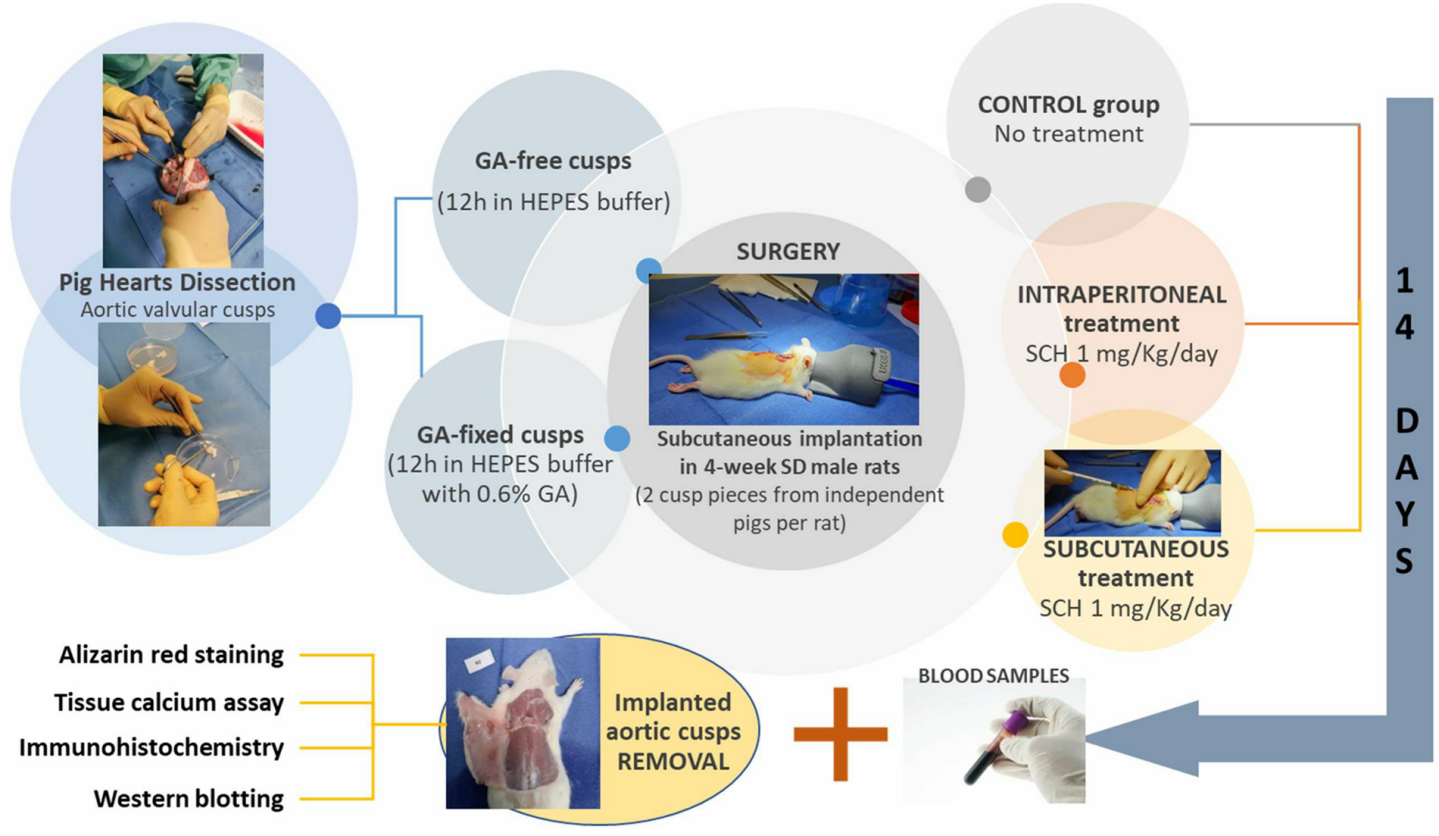
Schematic representation of the experimental design. GA, glutaraldehyde; HEPES, hydroxyethyl-piperazineethane-sulfonic acid buffer; IP, intraperitoneally; SC, subcutaneously; SCH, SCH527123 (a CXCR2 antagonist); SD, Sprague Dawley.

The design of the experimental protocol is illustrated in Figure 1. Four groups of 6 rats (12 independent porcine aortic valve cusp pieces each time) were compared: rats transplanted with GA-free cusps or GA-fixed cusps and those transplanted with GA-fixed cusps and treated with 1 mg/kg/day SCH5217123 (a CXCR2 antagonist, ref. 331-10731-5, RayBiotech) injected locally subcutaneously (SC) or intraperitoneally (IP). This dose was shown to be effective in various animal models of inflammation (13). Fourteen days later, blood sampling was made by cardiac punction under the xyphoid and the cusps were explanted and rinsed with PBS. Using micro-surgical instruments, the porcine cusps were unfolded and spread, then cut in 3 parts to be analyzed by alizarin red staining, calcium assay, immunohistochemistry and Western-blotting.

### 2.4. Blood cells count

The blood count was taken within one hour of the intracardiac puncture on an EDTA tube. The analysis was carried out on a XN-10 diagnostic system (Sysmex) and included red blood cells and white blood cells (lymphocytes, monocytes, neutrophils) counts.

### 2.5. Alizarin red staining

Pieces of porcine aortic valve cusps intended for the histological analyzes were fixed overnight with paraformaldehyde (PFA 4%) and two other nights with a 20% sucrose solution the first night and 30% the second in order to dehydrate the tissues. Then, they were included in OCT (Optimal cutting temperature compound, ref. KMA-0100-00A, Cell Path), snap-frozen in isopentane and stored at −80°C. A few days later, tissues were cut in a cryostat (LEICA CM 1950) in slices 12 μm thick. Several slides of the same valve were prepared and stored at −80°C so that they could be used later for alizarin staining as well as for immunostaining. On the day of staining, the slides were taken out of the freezer and then dried for 30 min at room temperature. Alizarin red solution (40 nM and pH 4.2, ref. A5533-25G, Sigma Aldrich) was gently applied to the slices. The excess dye was then removed and dehydration was carried out by incubation in a 100% acetone solution for 1 min and then in an acetone-toluene solution (1:1) for 1 min as well. Finally, 2 min incubation in a toluene solution was carried out before mounting with the DPX solution (ref. 0652, Sigma Aldrich). Photos of the valves were taken using the optical microscope (LEICA DM 2500).

### 2.6. Calcium assay

Pieces of porcine aortic valve cusps deposited in a 48-well plate were incubated overnight in a 0.6 N HCl solution, and then the content of tissue calcium was measured by the o-cresolphtalein complexone method. Briefly, a mixture containing 191 µg/ml of o-cresolphthalein and 2.33 mg/ml of 8-hydroxyquinoline with 0.5 M 2-Amino-2-methyl-1-propanol was prepared. The mixture was heated for 2 h at 65°C after covering it with parafilm to prevent evaporation. A volume of 25 µl of sample was taken from each well of the 48-well plate and then deposited in a new 96-well plate. A volume of 150 μl of the reaction mixture was added to each well. The plate was then shaken for 15 min at room temperature. Finally, the absorbance was read at 565 nm using a microplate reader and the amount of calcium was determined according to a standard curve of absorbance vs. calcium concentration. At the end of the assay, the valve pieces were dried at 37°C overnight to be weighed the next day on a precision balance. The calcium content of the valve cusp pieces was related to the tissue mass and, for each rat, the result of the 2 pieces of implanted valves was averaged.

### 2.7. Immunohistochemistry

Slices of porcine aortic valve cusps were unmasked with citrate for 15 min at 100°C, exposed to 0.3% H2O2 in PBS-1% BSA for 10 min at RT (to block endogenous peroxidase activity), and permeabilized for 1 h at RT with 0.15% Triton X-100 in PBS containing 1% BSA. Samples were then incubated overnight at 4°C with the primary antibodies anti-CD-3 (ref. A0452, Dako, dilution 1:100), anti-CD-11b (ref. ab8879, Abcam, dilution 1:1500), anti-CD-68 (ref. MCA341R, AbD Serotec, dilution 1:300), anti-CXCR2 (R&D Systems, MAB331, 1:50 dilution) or isotypes controls, in PBS with 1% BSA-0.1% Triton X-100. Samples were then rinsed three times in PBS with 1% BSA and incubated for 1 h at RT with the correspondent peroxidase-coupled secondary antibody (Cell signalling, 1:750 dilution) and thoroughly washed in PBS. Samples were then incubated with the peroxidase substrate from the Vector® NovaRED™ kit (Vector Labs, SK-4800) at RT until suitable staining developed. Finally, the coverslips were counterstained with haematoxylin solution and mounted on glass microscope slides using VectaMount™ mounting medium (Vector® H-5000). Sections were examined using an optical microscope (LEICA DM 2500). Quantitative analysis of the staining intensity was performed using ImageJ software.

### 2.8. Western blotting

Pieces of porcine aortic valve cusps were washed with PBS and then homogenized in NP-40 lysis buffer (25 mM Tris-HCl, 250 mM NaCl, 5 mM EDTA, and 1% SDS, pH 7.4) prepared with phosphatase and protease inhibitor cocktails, using gentleMACS tubes and a gentleMACS™ Dissociator. The supernatants were removed and the proteins quantified using a DC™ Protein Assay Kit (Bio-Rad) and diluted to equal concentrations. Protein samples were combined with 4X SDS sample buffer (8% SDS, 40% glycerol, 0.4% bromophenol blue, 5% β-mercaptoethanol, 240 mM Tris, pH 6.8), boiled 5 min at 95°C, and separated on 10% SDS-PAGE gels. After electrophoresis, proteins were transferred to nitrocellulose membranes (Ref. 10,600,002, Amersham,) using a Bio-Rad Trans-Blot Turbo Transfer System for 1 h. Membranes were blocked with 5% milk prepared in PBS 1%-Triton X-100 and then incubated with the primary antibodies anti-CXCR2 (Ref. MAB331, R&D Systems, 1:500 dilution), anti-CD68 (ref. MCA341R, AbD Serotec, dilution 1:300) or GAPDH (Ref. 2118, Cell signaling, 1:1,000 dilution) overnight at 4°C. After repeated washing, the membranes were incubated with the appropriate secondary antibody (dilution 1:1,000) in TBST with 5% milk for 1 h at room temperature and washed again. Then, the blots were developed using chemiluminescence reagent (Bio-Rad). The blots were viewed using a Chemidoc Imaging System (Bio-Rad) and the bands quantified using Image Lab software. The expression levels were corrected for loading differences by normalization against GAPDH.

### 2.9. Statistics

Data are reported as the mean values ± SEM. Student's *t*-test and the Mann–Whitney test were used for the statistical analysis of two groups of parametric and non-parametric data, respectively. For comparison of more than two groups, one-way ANOVA and Kruskal Wallis tests were performed for parametric data and non-parametric data, respectively. *P*-values were corrected for multiple comparisons by the Tukey's post-hoc method. They were two-sided and considered statistically significant if *P* < 0.05. Analyses were done using GraphPad Prism software (Version 7.0; GraphPad, San Diego, CA).

## 3. Results

### 3.1. Treatment of rats with SCH527123 reduces the calcification of GA-fixed cusps

**Figure 2:**
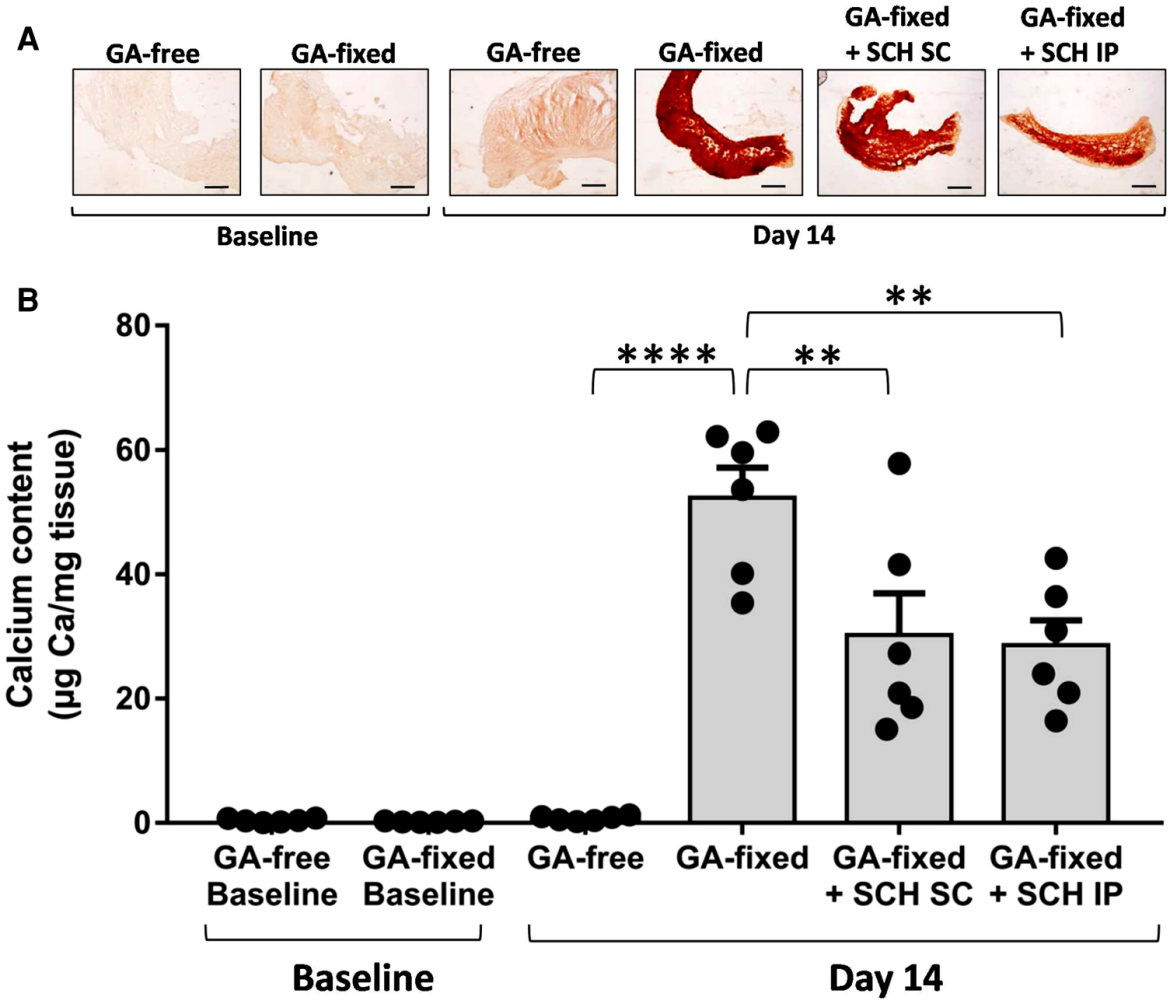
Treatment of rats with SCH527123 reduces the calcification of GA-fixed cusps. (**A**) Representative images of Alizarin Red staining in porcine aortic valve cusps, pre-incubated or not with glutaraldehyde (GA-free or GA-fixed respectively), before and after subcutaneous implantation for 14 days in rats treated or not with SCH527123, either intraperitoneally (IP) or subcutaneously (SC) around the xenograft. Scale bars: 50 μm. (**B**) Calcium content assayed by the o-Cresolphthalein Complexone method in GA-free and GA-fixed porcine aortic valve cusps, before and after subcutaneous implantation for 14 days in rats treated or not with SCH527123 IP or SC. Values are presented as the mean ± SEM of experiments performed in 6 rats per group with 2 cusps isolated from independent pig hearts per rat, and were compared by one-way ANOVA followed by Tukey's post-hoc test for multiple comparisons. **P < 0.01, ****P < 0.0001.

Results of Alizarin red staining and calcium assay are reported in the Figures 2A,B respectively. They show a strong 14-day calcification of implanted GA-fixed cusps in comparison to implanted GA-free cusps (52.3 ± 11.8 vs. 0.76 ± 0.4 µg Ca/mg tissues, *P* < 0.0001). To verify that the calcification was indeed produced during the two weeks following implantation, a comparison of the calcification was carried out between the group of valves not implanted on day 1 and those implanted (in the control rats) after explantation on day 14. This comparison confirmed the significant calcification of implanted GA-fixed cusps compared to those not implanted (52.3 ± 11.8 vs. 0.25 ± 0.1 µg Ca/mg tissues, *P* < 0.0001). Furthermore, the results of alizarin staining and calcium assay showed a significant reduction in calcification of implanted GA-fixed cusps in the group of rats treated with SCH527123 IP or SCH527123 SC compared to the untreated rats (28.6 ± 9.9 vs. 52.3 ± 11.8 µg Ca/mg tissues, *P* = 0.0013 or 30.2 ± 16.4 vs. 52.3 ± 11.8 µg Ca/mg tissues, *P* = 0.0030, respectively).

### 3.2. Treatment of rats with SCH527123 reduces blood leukocytosis

**Figure 3:**
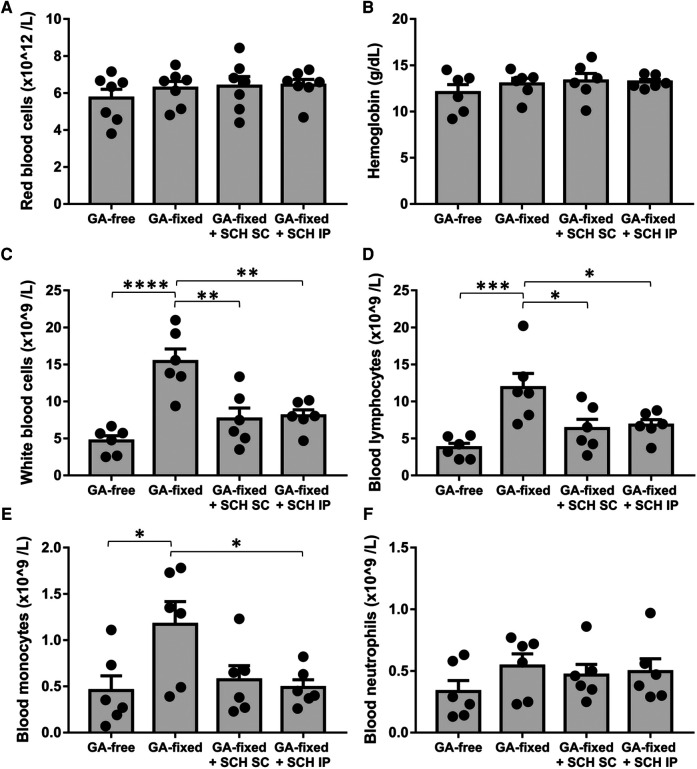
Treatment of rats with SCH527123 reduces blood leukocytosis. Blood count including red blood cells (**A**), hemoglobin level (**B**), white blood cells (**C**), lymphocytes (**D**), monocytes (**E**) and neutrophils (**F**) performed in rats, 14 days after the subcutaneous implantation of porcine aortic valve cusps pre-incubated or not with glutaraldehyde (GA-free or GA-fixed respectively), and treated or not with SCH (SCH527123) either intraperitoneally (IP) or subcutaneously (SC) around the xenograft. Values are presented as the mean ± SEM of experiments performed in 6 rats per group and were compared by one-way ANOVA followed by Tukey's post-hoc test for multiple comparisons. *P < 0.05, **P < 0.01, ***P < 0.001, ****P < 0.0001.

We analyzed the blood cell count in the different groups of rats at sacrifice (Figure 3). Results showed no significant variation in the red blood count and hemoglobin levels between conditions, but revealed a significant higher total white blood count and lymphocyte and monocyte counts in rats transplanted with GA-fixed cusps than in those transplanted with GA-free cusps (15.4 ± 0.8 vs. 4.7 × 10^9^/L, *P* < 0.0001; 11.9 ± 1.9 vs. 3.8 ± 0.6 × 10^9^/L, *P* = 0.0009 and 1.2 ± 0.3 vs. 0.5 ± 0.2 × 10^9^/L, *P* = 0.0034, respectively). These effects were significantly prevented in rats treated with SCH527123 administered IP (8.1 vs. 15.4 × 10^9^/L, *P* = 0.0027; 6.8 ± 0.7 vs. 11.9 ± 1.9 × 10^9^/L, *P* = 0.0422 and 0.5 ± 0.1 vs. 1.2 ± 0.3 × 10^9^/L, *P* = 0.0458) or SCH527123 administered SC for the white blood count (7.6 ± 1.5 vs. 15.4 × 10^9^/L, *P* = 0.0015) and the lymphocyte count (6.4 ± 1.2 vs. 11.9 ± 1.9 × 10^9^/L, *P* = 0.0239). Conversely, there was no significant change in the rat blood count of neutrophils between conditions.

### 3.3. Treatment of rats with SCH527123 reduces leukocytes infiltration in the GA-fixed cusps

**Figure 4:**
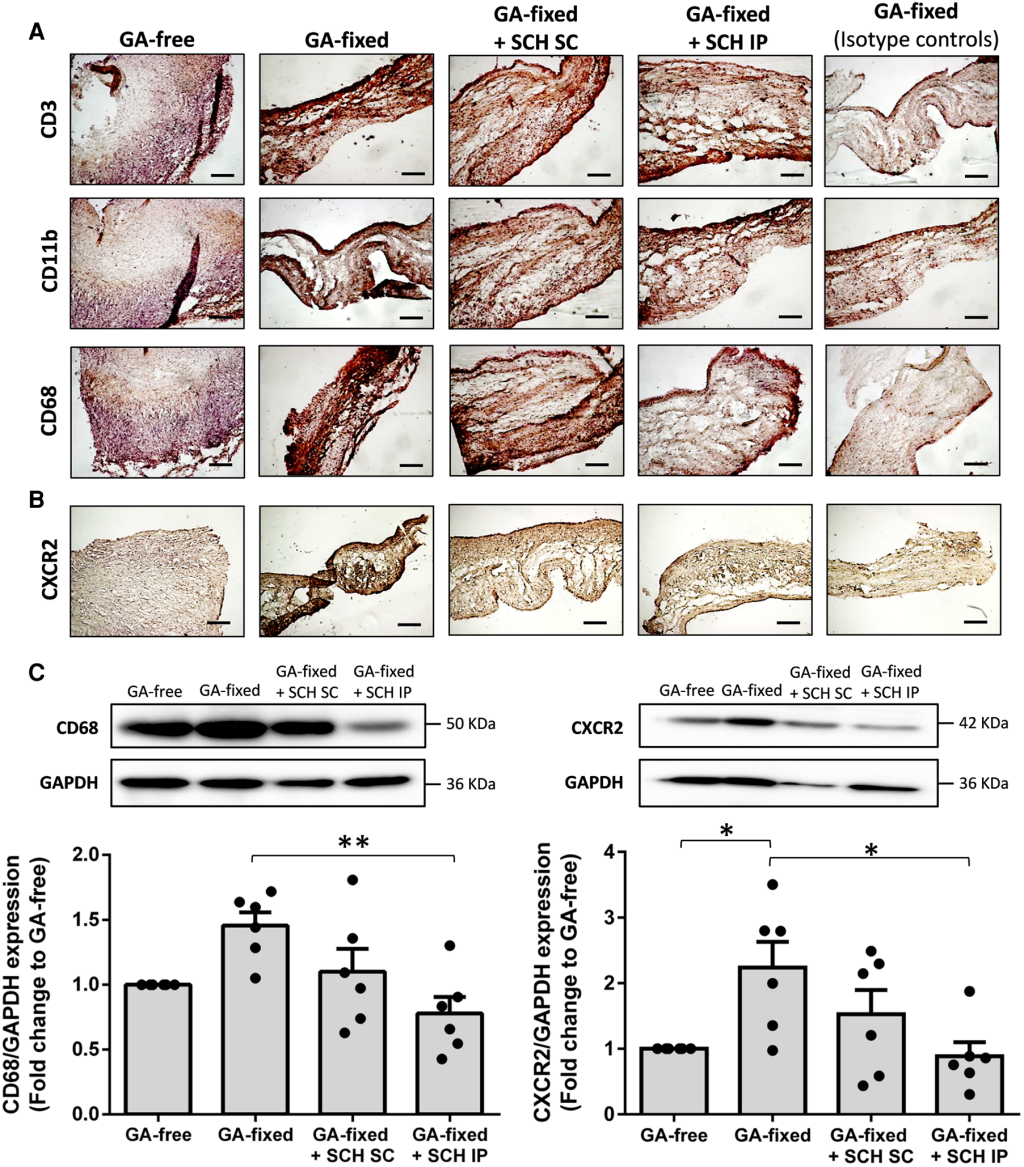
Treatment of rats with SCH527123 reduces leukocytes infiltration in the GA-fixed cusps. (**A**) Representative images of immuno-histological evaluation of the expression of CD3 (marker of lymphocytes), CD11b (marker of neutrophils and monocytes) and CD68 (marker of macrophages) in aortic valve cusps pre-incubated or not with glutaraldehyde (GA-free or GA-fixed respectively) after subcutaneous implantation for 14 days in rats treated or not with SCH (SCH527123), either intraperitoneally (IP) or subcutaneously (SC) around the xenograft. Control isotype IgG were used to assess the specificity of each antibody. Scale bars: 50 μm. (**B**) Representative images of immuno-histological evaluation of the expression of the C-X-C chemokines receptor type 2 (CXCR2) in GA-free and GA-fixed porcine aortic valve cusps, after subcutaneous implantation for 14 days in rats treated or not with SCH527123 IP or SC. Scale bars: 50 μm. (**C**) Representative western-blot analysis and quantification showing the expression of CD68 (left, weekly glycosylated) and CXCR2 (right) in GA-free and GA-fixed porcine aortic valve cusps, after subcutaneous implantation for 14 days in rats treated or not with SCH527123 IP or SC. Values are presented as the mean ± SEM of experiments performed in 6 rats per group and were compared by one-way ANOVA followed by Tukey's post-hoc test for multiple comparisons. *P < 0.05, **P < 0.01.

Representative images and quantitative results of immunohistochemistry analysis are provided in Figures 4A,B and the Supplementary Figure S1, respectively. They showed no significant difference in the staining of CD11b (a marker of neutrophils and monocytes) in GA-fixed cusps compared to GA-free cusps, after 14 days of subcutaneous implantation in rats (% of relative expression, 14.4 ± 9.2 vs. 9.3 ± 1.6 respectively, *P* = 0.5969). Conversely, a significant increase in the staining of CD3 (a marker of T-lymphocytes) and CD68 (a marker of macrophages) was shown in GA-fixed cusps compared to GA-free cusps (31 ± 11.9 vs. 12.7 ± 3.7 for CD3, *P* = 0.0072 and 28.1 ± 16.5 vs. 9.6 ± 5.4 for CD68, *P* = 0.0210, respectively), and these effects were significantly prevented in GA-fixed cusps explanted from rats treated with SCH527123 IP as compared to those explanted from non-treated rats (11.9 ± 8.1 vs. 31 for CD3, *P* = 0.0049 and 9.7 ± 3.3 vs. 28.1 ± 16.5 for CD68, *P* = 0.0213, respectively). In addition, CXCR2 staining was also increased in GA-fixed cusps compared to GA-free cusps (21.9 ± 6.3 vs. 6.6 ± 1.7, *P* < 0.0001, respectively), effects that were significantly reduced in the GA-fixed cusps explanted from rats treated with SCH527123 IP (10.6 ± 2.1 vs. 21.9 ± 6.3, *P* = 0.0006, respectively) or SC (13.2 ± 4.5 vs. 21.9 ± 6.3, *P* = 0.0076, respectively). Western blots also showed significant lower CD68 and CXCR2 expressions in GA-fixed cusps explanted from rats treated with SCH527123 IP as compared to GA-fixed cusps explanted from non-treated rats (in fold change to the GA-free cusps explanted from non-treated rats, 0.8 ± 0.1 vs. 1.5 ± 0.1, *P* = 0.0038 for CD68 and 0.9 ± 0.2 vs. 2.2 ± 0.4, *P* = 0.0177 for CXCR2, respectively) (Figure 4C).

## 4. Discussion

In this work, we showed that a treatment with the CXCR2 antagonist SCH527123 significantly prevented the calcification of porcine aortic valve cusps pre-incubated with GA and then implanted subcutaneously in rats for 14 days. This effect was associated with a reduction in the GA-induced rise of white blood cell count in rats and the infiltration of CD3-postive cells as well as CD68- and CXCR2-positive cells into the implanted porcine GA-fixed cusps.

CXCR2 is a transmembrane G-coupled protein receptor that binds with high affinity chemokines that contain a glutamic acid-leucine-arginine (ELR) motif, such as interleukin 8 in humans and CXCL1 and CXCL2 as well as the chemokine-like macrophage migration inhibitory factor (MIF-1) in humans and rodents (14). It plays an important role for the migration and activation of leukocytes of the innate immune system and the development of inflammatory diseases (15), including cardiovascular diseases (16). Indeed, CXCR2 was shown to have a major impact on macrophage accumulation in advanced atherosclerotic lesions (17–20). In the bioprosthetic heart valves explanted from patients with SVD, a significant increase in the immune cells infiltration associated with an elevated chemokines expression has been reported (12, 21, 22). These data corroborated the findings of Manji et al. who found evidence of immune rejection to GA-fixed xenografts in animal models, with calcification that was correlated with the intensity of macrophage infiltration (10, 23). Here, we report for the first time that pharmacologically blocking CXCR2 can reduce macrophages infiltration in GA-fixed xenografts and restrict the latter's calcification. Since CXCR2 is highly expressed by neutrophils and plays an important role in their functions (16), we cannot exclude that the preventive effect of antagonizing CXCR2 may have not been mediated through a reduction of neutrophils trafficking and/or activation. However, we did not evidence any change of the rats blood count of neutrophils between experimental conditions, on the contrary to monocytes whose increase levels in the GA-fixed condition was prevented by the treatment of rats with SCH527123. Moreover, the reduction of CD11b staining in the GA-fixed cusps under SCH527123 treatment was mild as compared to that of CD68 staining. This suggests that the treatment mainly led to a reduction in macrophage accumulation in xenografts, although this effect could result from an earlier broken cross-talk between neutrophils and monocytes/macrophages (24). Moreover, our results highlighted a significant rise in lymphocyte blood count as well as an increase in T-cells infiltration in the GA-fixed porcine cusps implanted for 14 days in rats. Accordingly, Manji et al. showed T cells (in addition to macrophages) invading GA-fixed xenografts by approximately 3 weeks post-transplant in rats, and Dahm et al. found a production of T cells directed specifically against GA-fixed pericardium implanted for from 2 weeks in rats, as well as a monocyte/macrophage infiltration at the interface between the xenograft and surrounding host tissue, a reaction that was comparable to a host-vs.-graft reaction (25). In our model, the treatment led to a strong inhibition of this reaction. We tested two modes of administration of the SCH527123: IP injection, which is a systemic mode of drug delivery, and local SC injections, which allow high drug concentrations around the xenograft. The results show that IP injections resulted in a much higher preventive effect on calcification and inflammation than SC injections, suggesting that the beneficial effect of the treatment may be more related to a systemic rather than local blocking of CXCR2. This supports the hypothesis that the xenograft calcification may have been limited through a systemic inhibition of the CXCR2-positive cells mobilization and/or homing. Clinically, the rate of SVD may be lower in transplant recipients undergoing immunosuppressive therapy (26, 27). Our work corroborates this finding showing that the CXCR2 antagonist SCH527123 was able to prevent by almost half the calcification of GA-fixed cusps through reducing the inflammatory response. The SCH527123 is an orally bioavailable compound already tested in humans for pulmonary inflammatory diseases and cancers (14), which strengthen the translational potential of our results. Moreover, we used young host animals known for their higher potential to develop calcification of the GA-fixed xenograft (28, 29). In clinical studies, the young patient age has been reported as an independent risk factor for early SVD (4); the age-related changes in the immune function may potentially affect SVD development (7). Here, we used a rat subcutaneous implant model as described by Mako et al. (30), who demonstrated significant levels of calcification as early as 7 days of implantation of GA-fixed tissues. We assessed calcification and inflammation after 14 days of implantation, which is a compromise between a sufficiently long time to have a frank calcification and nevertheless fast enough to evaluate the acute inflammatory response. Further analyzes will be needed to assess the time course of both pathophysiological events and treatment impact. Furthermore, this model does not take into account the hemodynamic constraints undergone by the valvular bioprosthesis after SAVR or TAVR in patients. Conversely, studies in juvenile sheep are also performed because experiments can be done in the systemic circulation. However, these experiments are complex, time-consuming, and expensive, on the contrary to those performed with the subcutaneous rat model, which can serve as a model for proof-of-concept studies testing both pathophysiology and therapeutic hypotheses (31). In our work, the macroscopic analysis of explanted GA-fixed cusps revealed the development of a neovascularization around a capsular contracture wrapping the tissue with a drop of blood inside (data not shown). This observation is consistent with clinical observations (32, 33), and supports the possibility for immune cells to have infiltrated the subcutaneous xenografts. Nevertheless, the subcutaneous rat model does not take into account the shear stress-induced inflammation experienced by the bioprostheses in the aortic position. Additional studies should therefore investigate how treatment by CXCR2 antagonism would restrict valve deterioration in the systemic circulation. Finally, although the role of inflammation in the development of calcification is well documented (7, 34), the mechanism by which the anti-inflammatory effect of SCH527123 has a beneficial effect on the calcification of GA-fixed xenograft requires further investigations.

## 5. Conclusion

The calcification of GA-fixed porcine aortic valve cusps, implanted subcutaneously in rats, is significantly prevented by antagonizing CXCR2 with SCH527123. The effect of SCH527123 might partly result from a reduction of the GA-induced T-cells and macrophage accumulation into the xenograft. These results thus open a new field for the development of preventive treatments of SVD in high risk patients, but warrant further studies to completely elucidate the involved mechanisms.

## Data Availability

The raw data supporting the conclusions of this article will be made available by the authors, without undue reservation.
