# Supplementary material for: Prevention by the CXCR2 antagonist SCH527123 of the calcification of porcine heart valve cusps implanted subcutaneously in rats
Source: Front Cardiovasc Med. 2023 Sep 15;10:1227589. doi: 10.3389/fcvm.2023.1227589 (PMC10540224; doi:10.3389/fcvm.2023.1227589)
Supplement: Supplementary file 1 [file Datasheet1.docx]

Prevention by the CXCR2 antagonist SCH527123 of the calcification of porcine heart valve cusps implanted subcutaneously in rats

Yuthiline Chabry, Kawthar Dhayni, Saïd Kamel, Thierry Caus, Youssef Bennis

Front. Cardiovasc. Med. 2023

Supplementary results.

**Supplementary Figure S1.**


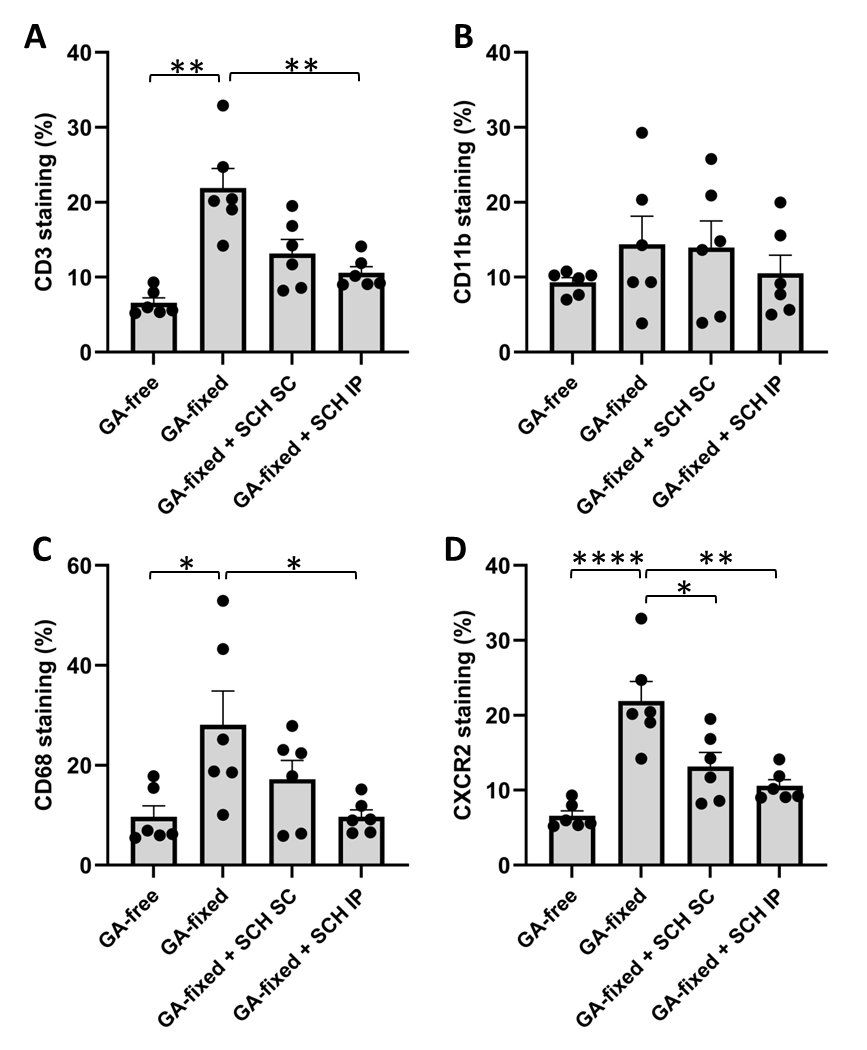


Immunohistochemistry quantification showing relative expression of CD3 (marker of T cells, Graph A), CD11b (marker of neutrophils and monocytes, Graph B), CD68 (marker of macrophages, Graph C) and the C-X-C expressing chemokines receptor type 2 (CXCR2, Graph D), in porcine aortic valve cusps pre-incubated or not with glutaraldehyde (GA-fixed or GA-free, respectively) after subcutaneous implantation for 14 days in rats treated or not with SCH (SCH527123), either intraperitoneally (IP) or subcutaneously (SC) around the xenograft. *P<0.5, **P<0.01, ****P<0.0001.
